# Functional prediction of de novo uni-genes from chicken transcriptomic data following infectious bursal disease virus at 3-days post-infection

**DOI:** 10.1186/s12864-021-07690-3

**Published:** 2021-06-19

**Authors:** Bahiyah Azli, Sharanya Ravi, Mohd Hair-Bejo, Abdul Rahman Omar, Aini Ideris, Nurulfiza Mat Isa

**Affiliations:** 1 Laboratory of Vaccine and Biomolecules, Institute of Bioscience, Universiti Putra Malaysia, 43400 Serdang, Selangor Darul Ehsan Malaysia; 2 Department of Veterinary Pathology and Microbiology, Faculty of Veterinary Medicine, Universiti Putra Malaysia, 43400 Serdang, Selangor Darul Ehsan Malaysia; 3 Department of Veterinary Clinical Studies, Faculty of Veterinary Medicine, Universiti Putra Malaysia, 43400 Serdang, Selangor Darul Ehsan Malaysia; 4 Department of Cell and Molecular Biology, Faculty of Biotechnology and Biomolecular Sciences, Universiti Putra Malaysia, 43400 Serdang, Selangor Darul Ehsan Malaysia

**Keywords:** *Gallus gallus*, RNA-sequencing, Transcriptomics, Infectious bursal disease virus, De novo, Bursa, Immune, Upregulated, Downregulated, Chickens

## Abstract

**Background:**

Infectious bursal disease (IBD) is an economically very important issue to the poultry industry and it is one of the major threats to the nation’s food security. The pathogen, a highly pathogenic strain of a very virulent IBD virus causes high mortality and immunosuppression in chickens. The importance of understanding the underlying genes that could combat this disease is now of global interest in order to control future outbreaks. We had looked at identified novel genes that could elucidate the pathogenicity of the virus following infection and at possible disease resistance genes present in chickens.

**Results:**

A set of sequences retrieved from IBD virus-infected chickens that did not map to the chicken reference genome were de novo assembled, clustered and analysed. From six inbred chicken lines, we managed to assemble 10,828 uni-transcripts and screened 618 uni-transcripts which were the most significant sequences to known genes, as determined by BLASTX searches. Based on the differentially expressed genes (DEGs) analysis, 12 commonly upregulated and 18 downregulated uni-genes present in all six inbred lines were identified with false discovery rate of q-value < 0.05. Yet, only 9 upregulated and 13 downregulated uni-genes had BLAST hits against the Non-redundant and Swiss-Prot databases. The genome ontology enrichment keywords of these DEGs were associated with immune response, cell signalling and apoptosis. Consequently, the Weighted Gene Correlation Network Analysis R tool was used to predict the functional annotation of the remaining unknown uni-genes with no significant BLAST hits. Interestingly, the functions of the three upregulated uni-genes were predicted to be related to innate immune response, while the five downregulated uni-genes were predicted to be related to cell surface functions. These results further elucidated and supported the current molecular knowledge regarding the pathophysiology of chicken’s bursal infected with IBDV.

**Conclusion:**

Our data revealed the commonly up- and downregulated novel uni-genes identified to be immune- and extracellular binding-related, respectively. Besides, these novel findings are valuable contributions in improving the current existing integrative chicken transcriptomics annotation and may pave a path towards the control of viral particles especially towards the suppression of IBD and other infectious diseases in chickens.

## Background

Infectious bursal disease (IBD) is an acute, highly contagious disease among chickens. It is one of the major factors leading to the drop in productivity and total economic loss to the poultry industry all over the world, irrespective of the country’s developmental stage [42]. IBD (also known as Gumboro disease) is commonly spread worldwide by two serotypes namely Serotype 1 and Serotype 2 [30, 43]. Serotype I consists of the sub-clinical (sc), classical virulent (cv) and very virulent (vv) types of strain reported to be responsible for disease manifestations seen in chickens [30], while Serotype 2 strains are more commonly found infecting turkey. These are serologically different than the IBD of chickens [18]. The IBD virus (IBDV) with the highest virulence characteristics was found infecting chicken despite the presence of a high level of maternal-derived antibodies in the host system, indicating the virus’s lethality. Thus, chicken mortality rates and bursal damage increase year by year [17, 25, 28, 39, 42], raising concerns globally. IBDV exhibits a selective tropism characteristic towards the B-cells of Bursa of Fabricius (BF) of the host [33]. Young chickens between the age of 3 to 6 weeks are the most susceptible to IBD. These are the specific range of time for the specialised haematopoiesis organ BF to be at its maximum rate of development and bursal follicles are filled up with immature B lymphocytes. IBD causes suppression of both humoral and cellular immunity in infected chickens. A severe IBD-viral immunosuppressed host chicken is susceptible to any viral, bacterial or parasitic secondary infection in its life that eventually leads to death.

The IBDV commonly enters the host organism (chicken) via the oral route and is transported to other tissues by phagocytic cells such as the resident macrophages in the blood circulation. The virus attacks the actively dividing B-cells which bear the IgM [37] and destroys the lymphoid follicles in BF, the circulating B-cells in the secondary lymphoid tissues such as GALT (gut-associated lymphoid tissue), CALT (conjunctiva), BALT (Bronchial), caecal tonsils and Harderian gland. Interestingly, unlike B-cells, T-cells of the infected host are not infected by the virus. Yet, they indirectly act as mediators for the pathogenesis. T-cells restrict the replication of the virus in BF cells during the early phase of infection by promoting bursal tissue damage and extending the time for tissue recovery through the release of cytokines [2, 43]. This self-defence mechanism eventually leads to further massive destruction and lesion of infected-host BF organ.

High-throughput RNA sequencing (RNA-Seq) is a powerful way to profile transcriptomic data with great efficiency and high accuracy. This fast-growing technology has been employed widely in various viral infections and diseases studies, especially in trying to understand the changes and effects on the host. It has the potential to reveal the dynamic alterations of the pathogen genome and the systemic changes in host gene expressions during the process of infection, which could help to uncover the pathogenesis of the infection by allowing observations of cell activities [4, 29, 31, 51]. Previously, transcriptomic analysis had been applied to compare the expressions of genes influenced by two different viral infections caused by influenza H5N8 and H1N, in mice of Park’s lab. The authors used this method to gain an in-depth understanding regarding the underlying genes involved in the pathogenesis of birds’ diseases by looking at their expression levels in two different samples, employing the case-control study method [31]. Besides, it is worth mentioning that we have analysed the poorly characterised genome-wide regulations of the immune responses of inbred chickens infected with vvIBDV in a previous study. Using RNA-Seq, transcriptome profiling of the bursa of infected chickens, we identified 4588 genes to be differentially expressed, with 1642 being downregulated genes and 2985 upregulated genes [11, 12]. The study reported bursal transcriptome profiles of differential expressions of pro-inflammatory chemokines and cytokines, JAK-STAT signalling genes, MAPK signalling genes and related pathways following vvIBDV infection. Although the RNA-Seq workflow analysis provided a concrete understanding of the transcriptomic activity of the bursa during vvIBDV infection at Day 3 p.i., there were approximately 10% unaligned reads to the NCBI *Gallus gallus* reference genome [13]. Hence, acting as a continuation of the previous research, this study aimed to analyse the differentially expressed genes in chickens of de novo assembled transcriptomes in response to vvIBDV infection. It would provide or new genes discoveries that could potentially aid in future therapeutic plans for better treatments against the disease to have healthy chicken populations in the poultry industry.

## Results

We had managed to cluster the unmapped reads from the previous study successfully. The clustered unmapped reads were then blasted against the BLAST query of Swiss-Prot and Non-redundant (NR) protein databases. However, out of the successfully clustered 10,828 reads, only 50–70% of the de novo reads had significant hits from both databases. To further answer questions on the potential pathogenesis of vvIBDV-infected bursa of chickens, we profiled differentially expressed genes of all six inbred lines using tools such as Cufflinks v2.0.2 and Cuffdiff v2.0.2 [48, 49]. Next, we observed the number of commonly upregulated and downregulated uni-genes which to be expressed in all lines were retrieved from the UpSetR [6], and again annotated against the Swiss-Prot and NR protein databases. Due to the presence of uni-genes without any hits against the two mentioned databases, the unknown uni-genes were tested using AUGUSTUS [46] and MATCH [20] in order to predict the Open Reading Frame (ORF) and Transcription Factor Binding Sites (TFBS), respectively. Seven out of the eight investigated unknown uni-genes had TFBS matches against the MATCH in-built database. However, only one each of the commonly upregulated and downregulated uni-genes were reported as having an ORF according to the Hidden Markov Model. Hence, we had also used the Weighted Gene Correlation Network Analysis R script [22] to outline the predicted function of the unknown sequences. By doing so, we were able to elucidate their potential functions by correlating the genes with no hits against genes with BLAST hits. Lastly, qRT-PCR quantitative validation test was performed on selected genes including upregulated and downregulated genes and a house-keeping gene, to validate our in silico RNA-seq outputs.

### RNA-Seq data analysis

**Fig. 1:**
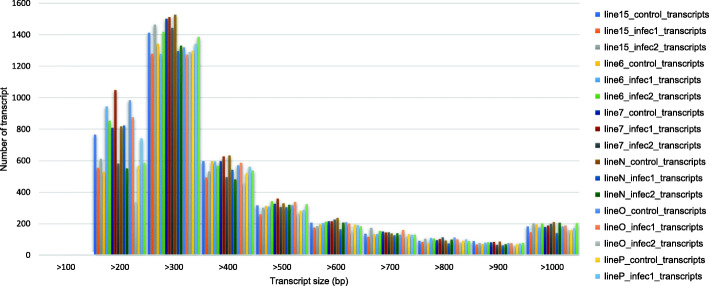
Size distribution of the assembled transcripts (bp) during the first stage in the Transcripts assembly and clustering method. The mentioned software managed to assemble unmapped reads into a set of assembled transcripts, ranging from 100 bp to more than 1000 bp. A great number of the generated assembled transcripts resided in the group size of 200-300 bp. All 18 transcriptomic data samples were colour-coded differently, as seen in the legend

The de novo transcript assembly of the unmapped reads was performed using Velvet [53] followed by Oases [40]. Initially, the K-mer size range of 45 to 71 was calculated for all 18 samples but only the K-mer size which yielded the highest N50 value for each sample was selected. This selection was done to maintain the quality of transcripts prior to de novo assembly. The final assembly was sorted according to size and those transcripts with bases less than 100 were discarded. As shown in Table 1, the shortest transcript size was 1,116,056 and the largest was 1,534,811. The N50 values were in the range of 382–454 with GC percentage > 62.79%. The average size of the transcripts ranged from 100 to 1000 bp and a large number of them fell into the range 200-300 bp as shown colour-coded to each sample respectively (Fig. 1).

**Table 1:** RNA-Seq data analysis mapping statistics on de novo assembly of unmapped reads

Sample	K-mer size	Unmapped reads (from reference assembly)		Transcripts assembled			
		Total	Paired	Number	Size	N50	GC%
Line 15 control	59	5,271,989	3,317,100	3801	1,401,130	409	63.76
Line 6 control	61	5,997,081	3,828,956	3454	1,334,830	433	64.51
Line 7 control	59	5,307,856	3,334,102	3957	1,443,431	405	63.38
Line N control	59	5,584,908	3,630,290	4077	1,520,131	413	63.65
Line O control	57	5,681,771	3,543,282	3904	1,423,493	412	63.92
Line P control	61	5,478,059	3,568,502	3323	1,253,231	423	63.96
Line 15 infec1	61	4,945,013	2,907,396	3181	1,171,966	406	64.22
Line 6 infec1	57	5,242,398	3,122,084	3830	1,394,985	409	63.59
Line 7 infec1	57	4,765,982	2,761,706	4285	1,534,811	400	64.16
Line N infec1	57	5,056,753	3,047,666	3538	1,244,627	382	63.98
Line O infec1	57	5,778,818	3,796,170	3800	1,410,323	424	64.36
Line P infec1	59	5,343,091	3,139,852	3608	1,329,105	407	64.38
Line 15 infec2	61	4,873,086	3,006,508	3659	1,404,734	441	63.34
Line 6 infec2	59	4,742,345	2,841,000	3940	1,476,426	430	63.65
Line 7 infec2	61	4,938,239	3,055,498	3583	1,382,526	445	63.51
Line N infec2	61	5,230,991	3,417,750	3412	1,336,894	454	62.99
Line O infec2	63	4,605,155	2,924,944	2911	1,116,056	423	63.89
Line P infec2	61	4,843,389	3,001,188	3525	1,364,545	432	62.79

A non-redundant set of uni-transcripts was generated from the 18 assembled transcripts. These results were from the pooling together and clustering of all the assembled transcripts until no new cluster was formed. Table 2 shows the mapping statistics report of the previously unmapped read transcripts from all six inbred chicken samples from the TIGR Gene Indices Clustering tool. A total of 10,828 uni-transcripts were produced with a total size of 5,577,804 bp, N50 of 713 bp and GC percentage of 62.05%.

**Table 2:** Results of transcript clustering using the TGICL software which generated a set of uni-transcripts. A total of 10,828 uni-transcripts were managed to be pooled together and clustered until no new cluster was formed

**Input**	Total number of transcripts from all samples	65,782
	Total size of transcripts from all samples	24,543,244b
	Transcripts N50 stats (bp)	382–454
	Transcripts GC%	62.79–64.51
**Output**	Total number of uni-transcripts	**10,828**
	Total size of uni-transcripts (bp)	5,577,804
	Uni-transcripts N50 stats (bp)	713
	Uni-transcripts GC%	62.05

### Complete Uni-transcript annotation from BLAST

The annotation was performed using a list of uni-transcript sequences in FASTA format. These uni-transcripts were searched against the NCBI NR database and the Swiss-Prot database by using BLASTX. The top 20 of the NR (protein) and the Swiss-Prot results respectively were analysed for Gene Ontology (GO) annotation. The overall BLAST results are presented in Table 3. Out of the 10,828 uni-transcript sequences, ~ 67% of them had at least one BLAST hit. More than 50% of the uni-transcripts received BLAST hits against both databases. The subjected uni-transcripts also had higher percentage of BLAST hits against the sense strand-template and a smaller value of hits against the antisense strand-template.

**Table 3:** Uni-transcripts annotation and BLAST analysis obtained from BLAST2GO. The generated uni-transcripts were subjected to BLAST2GO and BLAST against two databases, NR (protein) and Swiss-Prot databases. The uni-transcripts received > 50% BLAST hits against both mentioned databases. The subjected uni-transcripts also had a higher percentage of BLAST hits against the sense strand-template and a smaller value of hits against the antisense strand-template

Database	Number of uni-transcripts	Number of uni-transcript with ≥ 1 BLAST hit	%	Top BLAST hit			
				Sense	%	Antisense	%
NR (protein)	10,828	7291	67.33	6357	58.71	934	8.63
Swiss-Prot	10,828	6166	56.94	5598	51.70	568	5.25

**Fig. 2:**
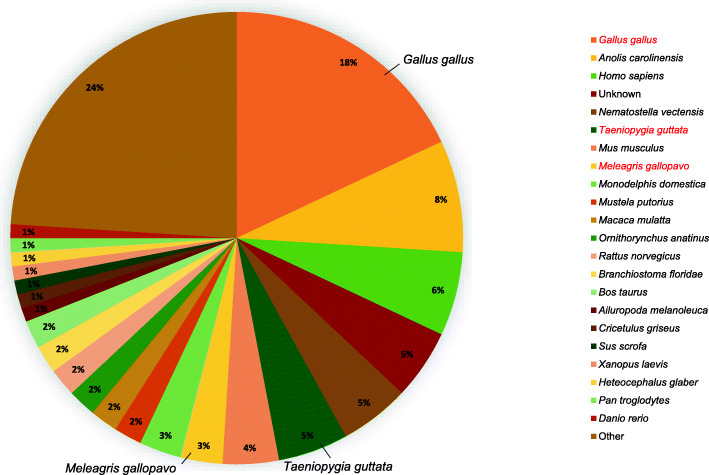
NR top species hit distribution of uni-transcripts obtained from BLAST2GO with respective percentages. Information provided from the pie chart were used to identify top species related to the uni-transcripts, according to the BLAST hits. A total of 23 species was reported but only three of those mentioned in the legend were bird-related species; *Gallus gallus*, *Taeniopygia gutata* and *Meleagris gallopavo* (highlighted in red)

The NR top species hit distribution (Fig. 2) revealed that among the uni-transcript sequences with BLAST hits, 18% belonged to *Gallus gallus;* annotated as the species with the maximum number of hits among the uni-transcript sequences. Interestingly, out of the top 23 species hit distribution annotated, *Taeniopygia guttata* (5%) and *Meleagris gallopavo* (3%) were the only two hit species related to birds. This suggested that the rest of the sequences could potentially be novel sequences against *Gallus gallus* or that they could have resulted due to some sequencing errors.

### Identification of differentially expressed (DE) Uni-genes

To understand the gene expression in the control versus the IBDV-infected condition, DE gene analysis was carried out. The expressions of the transcriptomes are presented in Table 4, where the numbers of sequences with FPKM values > 0 and > 1e-5 threshold along with their percentage values are displayed. Meanwhile, Table 5 shows the numbers of sequences significantly upregulated and downregulated, and the uniquely up- and downregulated ones for each sample during the infected and control states. After calculations, approximately, 85% (now called uni-genes) out of the 10,282 uni-transcripts were seen to be differentially expressed. Relatively, 130–569 uni-genes of the six inbred lines were suggested to be responsive towards IBDV-infection, where Line O had the smallest DE number and Line 15 had the largest DE number. The total number of sequences that were differentially expressed was 1697. However, this result contained redundant sequences. Upon the removal of the redundant sequences in the uni-transcripts by mapping previously unmapped reads against the uni-transcripts, the new total number of uni-gene sequences uniquely differentially expressed was now 618.

**Table 4:** Expression analysis of uni-transcripts in FPKM and its percentage respective to all transcriptome data obtained from Cufflink. Only uni-transcripts with FPKM cut-off value >1e-5 were reported in the table

Sample	Total number of uni-transcripts	Number of non-zero FPKM uni-transcripts	%	Number of uni-transcripts with FPKM > 1e-5	%
Line 15 control	10,828	8961	82.76	8961	82.76
Line 6 control	10,828	9108	84.12	9108	84.12
Line 7 control	10,828	9041	83.50	9041	83.50
Line N control	10,828	9090	83.95	9090	83.95
Line O control	10,828	8974	82.88	8974	82.88
Line P control	10,828	8866	81.88	8866	81.88
Line 15 infec1	10,828	8865	81.87	8865	81.87
Line 6 infec1	10,828	9028	83.38	9028	83.38
Line 7 infec1	10,828	9019	83.29	9019	83.29
Line N infec1	10,828	8856	81.79	8856	81.79
Line O infec1	10,828	8930	82.74	8930	82.74
Line P infec1	10,828	8804	81.31	8804	81.31
Line 15 infec2	10,828	9234	85.28	9234	85.28
Line 6 infec2	10,828	9241	85.34	9241	85.34
Line 7 infec2	10,828	9258	85.50	9258	85.50
Line N infec2	10,828	9246	85.39	9246	85.39
Line O infec2	10,828	9289	85.79	9289	85.79
Line P infec2	10,828	9145	84.46	9145	84.46

**Table 5:** Differentially expressed uni-transcripts (IBDV-infected versus Control) produced by Cufflink, for all six inbred lines. Uniquely up- or downregulated uni-transcripts in the samples were uni-transcripts screened to be only present in only one sample

	Line 15	Line 6	Line 7	Line N	Line O	Line P
**Upregulated in infected samples**	359	136	177	102	74	222
**Downregulated in infected samples**	206	96	94	47	56	123
**Uniquely in infected samples**	3	0	1	0	0	0
**Uniquely in control samples**	1	0	0	0	0	0
**Total**	569	232	272	149	130	345

### Identification of commonly DE Uni-genes

**Fig. 3:**
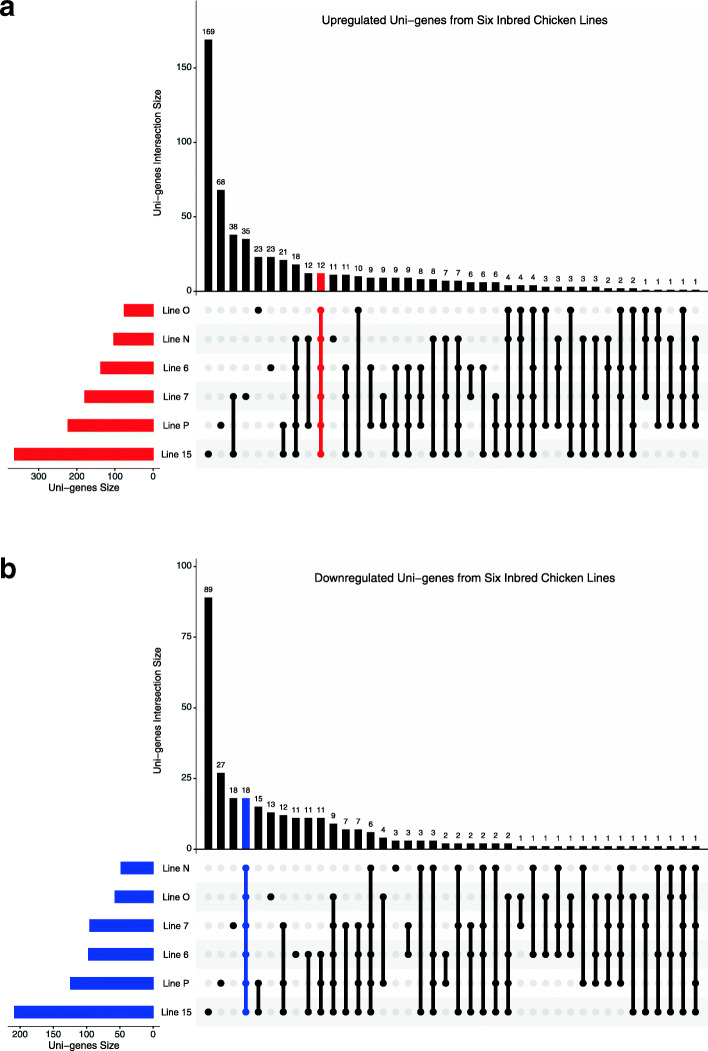
UpSet R plot representing (**a**) upregulated and (**b**) downregulated uni-genes. The lines in red and blue represent the up- or downregulated uni-genes in all six lines in IBDV-infected chickens at 3 days p.i. These were then called as commonly up- or down-regulated uni-genes. The upper bar chart shows the uni-genes that intersected in different combinations of inbred lines, the bottom right exhibits the combination of inbred lines and the bottom left shows the uni-genes size per inbred line

R package UpSetR [6] was used to plot the intersection size accordingly to every possible combination of inbred lines. The input was a tabulated 618 short-listed number of uni-gene sequences screened to be significantly differentially expressed with *p* < 0.05 along all six lines of inbred chickens. The numbers displayed represented the number of sequences which appeared to be upregulated (Fig. 3a) and downregulated (Fig. 3b) in all the line combinations. Among the reported DE uni-genes, 12 commonly upregulated (emphasised in red) and 18 commonly downregulated (emphasised in blue) uni-genes were observed to be expressed across all lines irrespective of their genetic backgrounds. This was an interesting finding as it might provide a deeper understanding at the molecular level of IBDV-infection in chickens at the chicken’s Bursa of Fabricius especially in elucidating the pathophysiology of the disease.

### BLAST2GO of commonly DE Uni-genes analysis

**Fig. 4:**
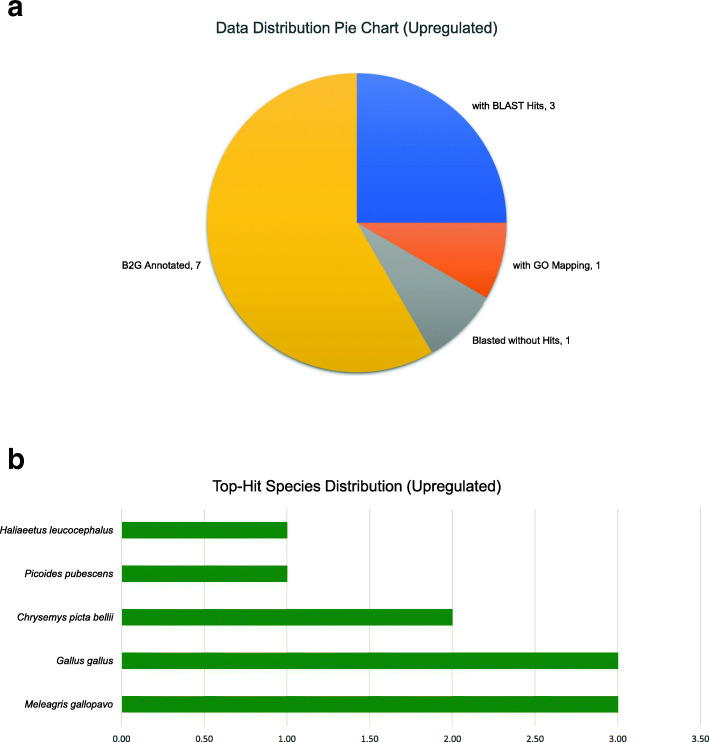
BLAST2GO results of 12 upregulated uni-genes sequences. The information obtained was displayed accordingly to BLAST hits of the subjected upregulated sequences such as (**a**) data distribution pie chart and (**b**) species distribution of the top hits. Three sequences received no BLAST hits, suggesting possible novel gene sequences. Furthermore, rather than *Gallus gallus*, *Meleagris gallopavo* was reported to be the top species with the highest BLAST hits

**Fig. 5:**
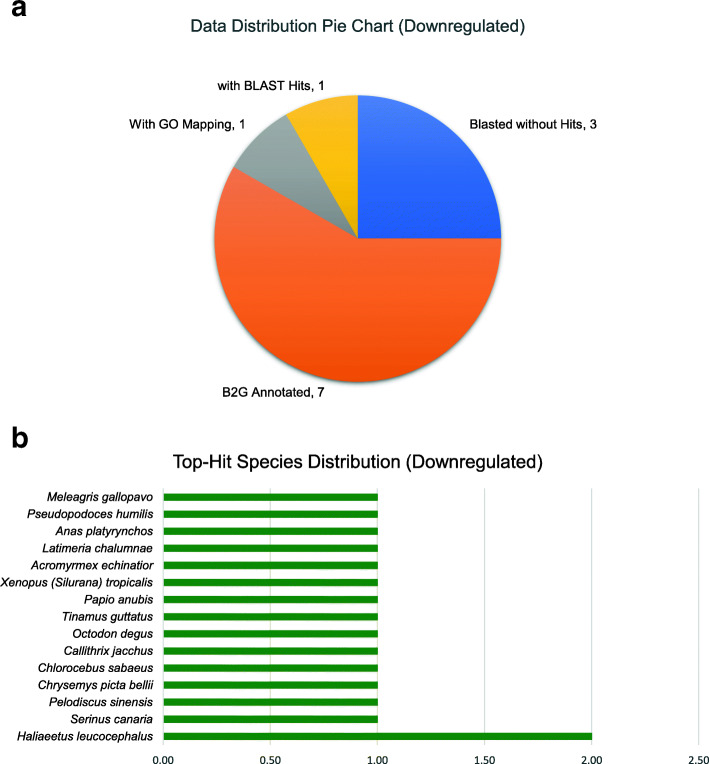
BLAST2GO results of 18 downregulated uni-genes sequences. The information obtained was displayed accordingly to the BLAST hits of the subjected downregulated sequences such as (**a**) data distribution pie chart and (**b**) species distribution of the top hits. Five sequences received no BLAST hits. Interestingly, *Gallus gallus* was not in the top-hit species distribution

The commonly upregulated and downregulated uni-genes from the gene intersection analysis were subjected to BLAST2GO, to find gene information by matching sequence with related existing gene annotations in the BLAST database. Out of the 12 upregulated uni-genes, there were seven sequences with annotation, one with just BLAST hit, one with GO mapping and three with no BLAST hit (Fig. 4a). Similarly, Fig. 5a presents the data distribution for the downregulated uni-genes. There were 13 sequences with BLAST hits, and five downregulated sequences out of the 18, which did not have any homologue in the NCBI NR database. According to Fig. 4b, only three out of the 12 upregulated uni-gene sequences were annotated to belong to *Gallus gallus.* The rest of the DE uni-gene sequences belonged to other bird species like *Meleagris gallopavo* (Wild Turkey), *Chrysemys picta* (Painted Turtle), *Haliaeetus leucocephalus* (Bald Eagle) and *Picoides pubescens* (Downy Woodcutter). On the other hand, none of the downregulated uni-genes sequences was highlighted to have hits to *Gallus gallus* (Fig. 5b), but acquired two hits against *Haliaeetus leucocephalus* (Bald eagle) while only one hit was on the rest of the species distribution.

Table 6 and Table 7 list the up- and downregulated uni-gene sequences with the respective top BLAST hit along with its functional description, percentage similarity and E-value. All upregulated uni-genes with hits had similarity scores of more than 70% while the downregulated uni-genes were with hits similarity score ranging from 48 to 100%. Hits of uni-genes with high similarity scores and significant E-values provide us with in-depth information regarding sequences novel against the *Gallus gallus* reference genome. Surprisingly, according to the BLAST assessments, there were three upregulated and five downregulated uni-gene sequences that did not have any significant homologue in the database.

**Table 6:** List of 12 upregulated uni-genes sequences with the corresponding BLAST hits results, ranked according to the similarity score %. The respective BLAST hits description, similarity score and E-value were also reported. Nine uni-gene sequences were with hits from the BLAST database, while three sequences had no BLAST hit

Upregulated Uni-genes	BLAST Hit Description	Similarity Score (%)	E-value
1_CL1782Contig1	mucin-13 isoform XI	100	2.14E-27
1_CL2243Contig1	extracellular fatty acid-binding	100	1.76E-106
lineP_ifc1_Lc_736_T_1/1_C_1.000_L_349	protein s100-a10	100	4.04E-42
1_CL175Contig1	ccaat enhancer-binding protein delta	97	2.52E-47
1_CL2788Contig1	extracellular matrix protein 1	97	2.91E-60
lineN_ifc2_Lc_670_T_2/3_C_0.800_L_748	homeobox 1	96	2.25E-47
1_CL1663Contig1	ccaat enhancer-binding protein delta	96	1.08E-40
1_CL1597Contig1	interleukin-18 binding protein	90	4.79E-38
1_CL1663Contig2	ccaat enhancer-binding protein delta	71	1.22E-59
1_CL12Contig16	**--NA--**		
1_CL2624Contig1	**--NA--**		
1_CL41Contig6	**--NA--**

**Table 7:** List of 18 downregulated uni-gene sequences with the corresponding BLAST hits results, ranked according to the similarity score %. The respective BLAST hits description, similarity score and E-value were also reported. There were 13 uni-gene sequences with hits from the BLAST database, while five sequences had no BLAST hit

Downregulated Uni-genes	BLAST Hit Description	Similarity Score (%)	E-value
1_CL1624Contig1	nicotinamide riboside kinase 2	100	2.72E-14
1_CL2708Contig1	cerebellar degeneration-related protein 2	100	7.71E-25
1_CL2738Contig1	sterile alpha motif domain-containing protein 11 isoform ×2	100	3.64E-88
1_CL2743Contig1	GMP reductase 1	100	4.52E-27
1_CL3191Contig1	e3 ubiquitin-protein ligase uhrf1	100	1.07E-44
1_CL3404Contig1	e3 ubiquitin-protein ligase uhrf1	100	1.14E-44
2_CL441Contig1	ubiquitin-conjugating enzyme e2c	97	2.66E-60
1_CL1209Contig1	RNA-binding protein 38	95	1.96E-51
1_CL457Contig2	aurora kinase b	84	0
1_CL404Contig1	DNA replication licensing factor mcm7	82	4.52E-167
1_CL7Contig4	DNA-directed rna polymerase ii subunit rpb1	52	5.79E-04
lineN_ctrl_Lc_456_T_1/1_C_1.000_L_725	cell surface protein	49	3.37E-12
1_CL2740Contig1	b-cell receptor cd22 isoform ×2	48	2.45E-55
1_CL2484Contig1	**--NA--**		
1_CL1576Contig1	**--NA--**		
1_CL1679Contig3	**--NA--**		
1_CL2766Contig1	**--NA--**		
1_CL2572Contig1	**--NA--**		

### Gene ontology (GO) enrichment analysis of commonly DE Uni-genes

**Fig. 6:**
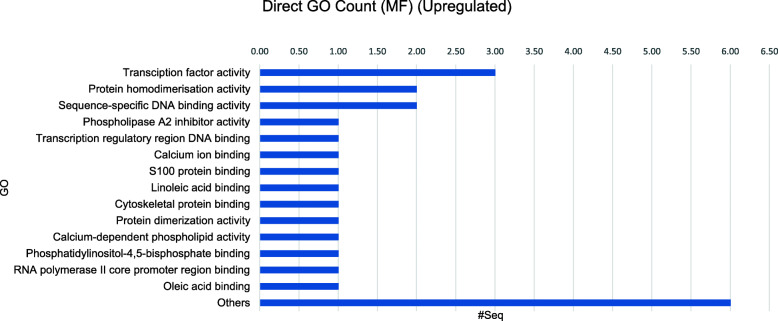
GO terms domain categories of the 9 commonly DE upregulated uni-genes

**Fig. 7:**
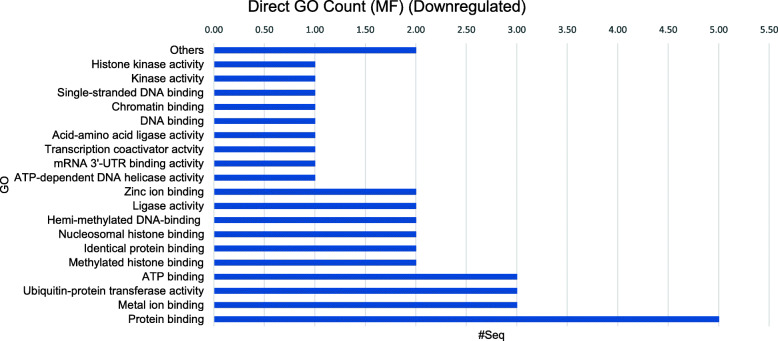
GO terms domain categories of the 13 commonly DE downregulated uni-genes

The BLAST2GO tool also produces output information regarding the functional annotations and related GO term domain categories hits distribution. The functional annotations of uni-genes sequences with BLAST hits of the upregulated and downregulated sequences are displayed in Figs. 6 and 7, respectively. The GO terms domain categories distribution for the molecular functions (MF) is displayed in both figures for comparison.

The top 3 annotated MF of the commonly upregulated uni-genes were involved in the transcription factor activity, protein homodimerization activity and sequence-specific DNA binding transcription factor activity (Fig. 6). Meanwhile, the top 3 MF for the commonly downregulated uni-gene sequences were with protein binding, metal ion binding and ubiquitin-protein transferase activities (Fig. 7). The annotations of the commonly DE uni-genes identified showed a decrease of bursal cells activities in cellular signalling and an increase of differentiation activities. Briefly, the overall results revealed that the common functional differences between the IBDV-infected and the control condition were related either to immune, cellular signalling or cell proliferation. Both results might help in elucidating a clearer picture regarding the physiological condition of Bursa of Fabricius cells following IBDV infection at 3-days post-infection.

### Gene prediction of commonly DE Uni-genes with no BLAST hit

Gene prediction obtained by using AUGUSTUS [46] was carried out due to the presence of common DE uni-genes with no BLAST hits against the BLAST database. The ORF of the input uni-gene sequences would be detected by the AUGUSTUS algorithm which would also predict the gene coding region by finding the START codon and the end sequence by searching for the nearest STOP codon.

Accordingly, in this study, only one predicted ORF sequence was produced by AUGUSTUS for both the commonly upregulated sequences and the downregulated sequences (Table 8). The lengths of both the predicted ORF sequences were bp length of 484 and 588, respectively for the upregulated and downregulated sequences listed. This result suggested that the other two unknown upregulated and the four unknown downregulated uni-genes sequences that did not have ORF prediction results had high probabilities to be parts of bigger sequences that we did not manage to assemble previously. It should be pointed out that it might also suggest that the sequences did not have the sites that aid in the prediction of the ORF. Nevertheless, the predicted ORFs output by AUGUSTUS indicated that there could be a novel gene that had not been identified before in the annotated transcriptomics of *Gallus gallus*.

**Table 8:** AUGUSTUS results showing one sequence from both the up- and the downregulated list of sequences that did not have BLAST results. The table shows the length of the sequence, the start and stop positions of the predicted open reading frame (ORF) coding region and the potential protein sequence

Uni-gene	Length	Start	Stop	Amino Acid Sequence
1_CL12Contig16 (upregulated)	588	1	501	MALQRSMKAEEEEEEEEEEAQLAMALQRSIREKEKEEEEEEVRLVMTLQRSIKEKEKEEEEEEVRLAMALQRSMKEEEEEEEEEEEAQLAMALQRSIREKKEEEEEEEEAQLAMALQRSMKEEEEEEEKEVQLAMALQRSMKEEEEEEEEEEEAQLAMALQRSIREK
1_CL2484Contig1 (downregulated)	484	1	409	GGGEEEEEEEEEEEEEGYEQPDSDSNTDGYENEGGAAPSQSSAGSYENDPNSTPASDGPPTPTDPTVLSPGAANLITGLQRALLAAQRWDGRSDGSAGSQPYEEMGGTKRAALRRDGTEDDAGSYENMAGAELTP

### Transcription factor binding sites analysis

TFBS analysis was conducted as one of the steps to further elucidate the characteristics of our de novo uni-genes with no BLAST hits. Using the geneXplain MATCH program [20], the fasta file of three upregulated and five downregulated unknown uni-genes were inserted as input. Among all the eight commonly differentially expressed uni-genes, only one (1_CL2766Contig1) uni-gene returned with no information or match against the TRANSFAC 6.0 database [52] (Table 9). All seven matches had a core-score of > 0.95 with a matrix-match score of > 0.93. In brief, seven out of the eight novel uni-genes proposed in this study had essential regions which allowed regulation of gene expression activities. These reported features provided concrete evidence to consider our novel uni-genes as complete functional DNA sequences.

**Table 9:** MATCH program output results showing predicted transcription factors binding site (TFBS) of commonly differentially expressed uni-genes with no BLAST hits, according to the uni-genes DNA sequences. The table displays the core match score, matrix match score, predicted transcription factor with its respective TFBS DNA sequence and UniProt ID. Only 1_CL2766Contig of the downregulated uni-genes was reported with no match against the TRANSFAC database

Uni-gene	DE	Core match	Matrix match	Sequence	Transcription Factor	Uniprot ID
1_CL12Contig16	Up	1	0.931	ccgtaCTTCCtcttcc	Elk-1	P19419
1_CL2624Contig1	Up	1	0.965	ccgccgGCTTTaatt	Barbie box	n/a
1_CL41Contig6	Up	1	1	tgACGTCa	c-Jun / AP-1	P05627
1_CL1576Contig1	Down	0.997	0.893	cctcctttctCTTCT	HSF1	P10961
1_CL1679Contig3	Down	1	1	tGACGTta	c-Jun / AP-1	P05627
1_CL2484Contig1	Down	1	1	cgtgACCCC	CF-1 / USP	P20153
1_CL2572Contig1	Down	0.953	0.983	gttCCGGAacgttct	HSF1	P10961
1_CL2766Contig1	Down	**–**	**–**	**–**	**–**	**–**

### WGCNA of commonly DE Uni-genes analysis

In this analysis, we had determined to only analyse four unknown downregulated uni-genes. Consequently, only three commonly upregulated and four downregulated uni-genes sequences that did not have any hit in BLAST were assigned to specific modules based on their correlation coefficients calculated using the WGCNA R package in order to further annotate the de novo uni-genes. The functional prediction of the unknown uni-genes sequences namely 1_CL12Contig16, 1_CL2624Contig1, 1_CL41Contig6 in the upregulated and 1_CL1576Contig1, 1_CL1679Contig3, 1_CL2484Contig1 and a 1_CL2766Contig1 in the downregulated list of sequences was performed by clustering these unknown uni-gene sequences with sequences with BLAST hits according to their related expression patterns.

### Upregulated gene network

**Fig. 8:**
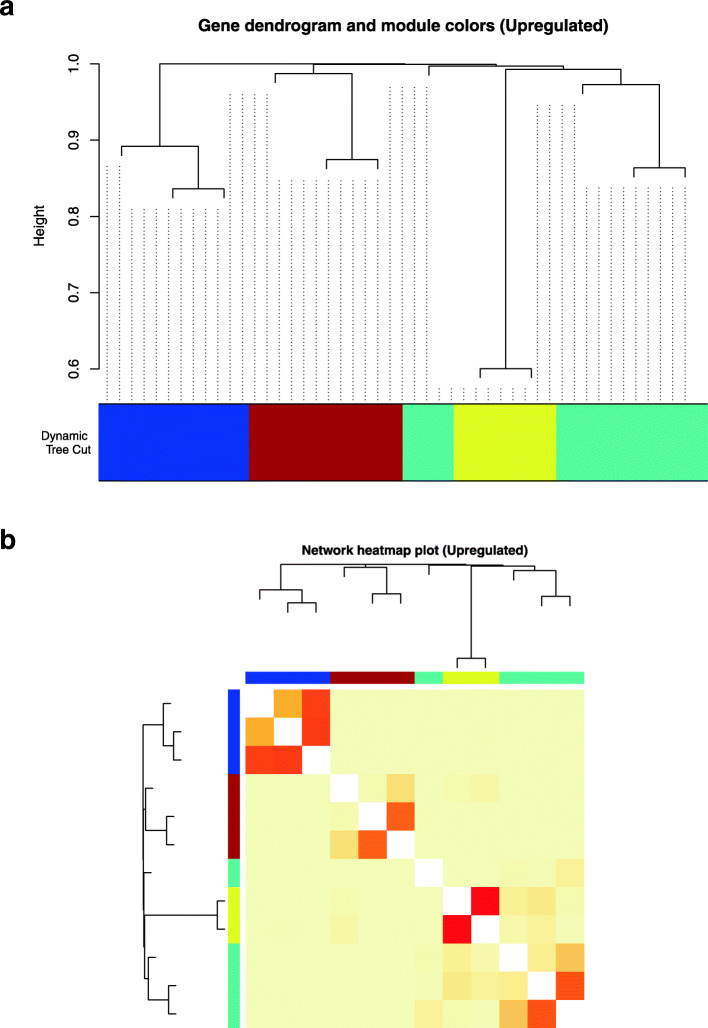
WGCNA results regarding function predictions of upregulated uni-gene sequences with no BLAST hits. Using the built-in tools of WGCNA, three unknown upregulated uni-gene sequences managed to be clustered to the available coloured-module brown and blue. The Figure above shows (**a**) gene dendrogram and module colours, and (**b**) the network heatmap plot of the upregulated uni-genes

After subjecting them to WGCNA, 12 upregulated uni-genes with unknown functions were grouped into four colour-coded modules: Yellow, Brown, Blue and Turquoise. The three unknown uni-genes with no BLAST hit were found to belong to only two of the four modules (Table 10). The module dendrogram of the upregulated sequences (Fig. 8a and b) displayed the unknown sequences which belonged to the Blue and the brown modules. Interestingly, the Blue module contained the unknown sequence 1_CL12Contig16 and the known sequences that had BLAST hits of mucin-13 isoform × 1 (100% *Gallus gallus*) and extracellular matrix protein 1 (100% Wild Turkey). Meanwhile, the Brown module in the upregulated list had two unknown uni-gene sequences 1_CL2624Contig1 and 1_CL41Contig6 associated with the known sequence whose BLAST hit was interleukin-18-binding protein (90% Wild Turkey).

**Table 10:** Modules of upregulated uni-gene sequences produced by the WGCNA R tool. Three unknown functions of upregulated uni-genes were subjected to WGCNA and they have clustered accordingly to the available colour-coded modules. The table provides information on possible functional annotations of the unknown upregulated sequences by comparing against sequences with known functional annotations

Sequence with No BLAST Hit	Module Colour	Sequence with BLAST Hit	Annotation
**1_CL12Contig16**	Blue	1_1782Contig1	mucin 13 isoform xi
		1_2788Contig1	extracellular matrix protein
**1_CL2624Contig1**	Brown	CL1597Contig1	interleukin-18 binding protein
**1_CL41Contig6**

### Downregulated gene network

**Fig. 9:**
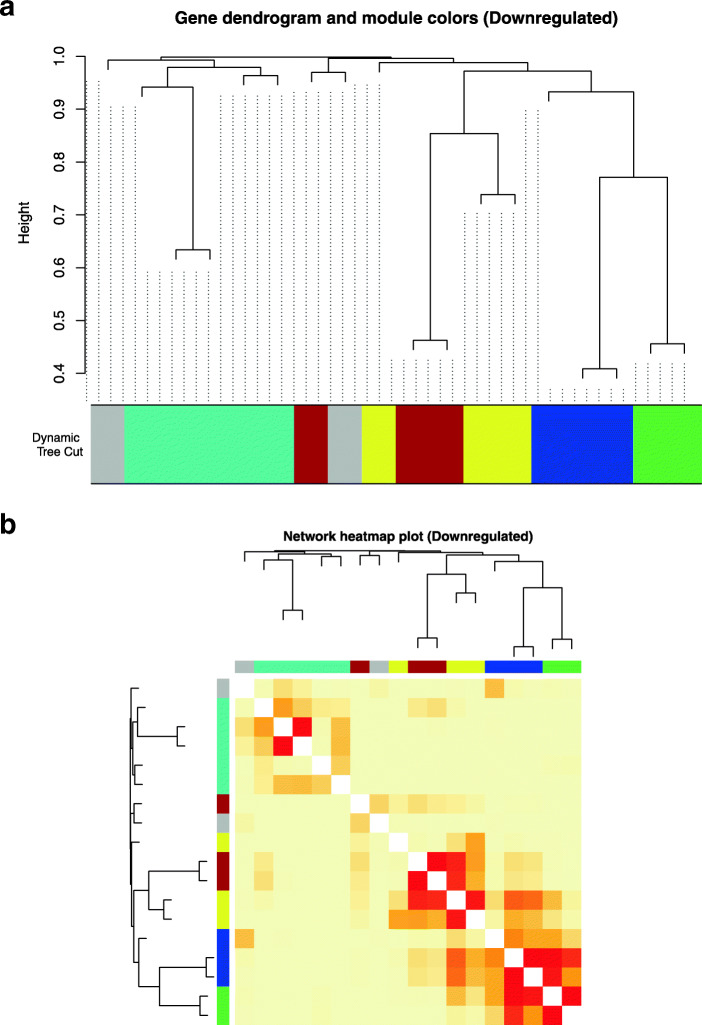
WGCNA results regarding function prediction of downregulated uni-gene sequences with no BLAST hits. Using the built-in tools of WGCNA, four unknown downregulated uni-gene sequences managed to be clustered to the available coloured-module turquoise, brown and blue. The Figure above shows (**a**) gene dendrogram and module colours cluster, and (**b**) the network heatmap plot of the downregulated uni-genes

Accordingly, 18 downregulated unknown uni-genes were arranged into three coloured modules after merging closely related modules: Turquoise, Brown and Blue (Fig. 9a and b). Out of these three modules, sequences with no BLAST hits belonged to modules where there were sequences that had annotations (Table 11). The Blue module contained unknown sequence 1_CL2484Contig1 and the BLAST hits of the known sequences were nicotinamide riboside kinase 2 (100% Domestic Turkey) and ubiquitin-conjugating enzyme e2c (97% Ground tit). The Brown module contained unknown 1_CL1679Contig3 and 1_CL2766Contig1 that were associated with the known sequence having the BLAST hit of cell surface protein (49% White-throated tinamou). Finally, the Turquoise module contained the unknown sequence 1_CL1576Contig1 along with the known sequences with their corresponding BLAST hits, RNA-binding protein 38 (95% Rodent), cerebellar degeneration-related protein 2 (100% Atlantic canary), e3 ubiquitin-protein ligase uhrf1 (100% Sea Eagle) and DNA replication licensing factor mcm7 (82% Frog).

**Table 11:** Modules of downregulated uni-gene sequences produced by the WGCNA R tool. Only four unknown functions of downregulated uni-genes were subjected to WGCNA and they have clustered accordingly to the available colour-coded modules. The table provides information on possible functional annotation of the unknown upregulated sequences by comparing against sequences with known functional annotations

Sequence with No BLAST Hit	Module Colour	Sequence with BLAST Hit	Annotation
**1_CL1576Contig1**	Turquoise	1_CL1209Contig1	RNA-binding protein 38
		1_2708Contig1	cerebellar degeneration-related protein 2
		1_3404Contig1	e3 ubiquitin-protein ligase uhrf1
		1_404Contig1	DNA replication licensing factor mcm7
**1_CL1679Contig3**	Brown	lineN_ctrl_Lc_456_T_1/1_C_1.000_L_725	cell surface protein
**1_CL2766Contig1**			
**1_CL2484Contig1**	Blue	1_CL1624Contig1	nicotinamide riboside kinase 2
		2_CL441Contig1	ubiquitin-conjugating enzyme e2c

### Quantitative validation using qRT-PCR

To verify the accuracy and reproducibility of the RNA-Seq prediction produced in silico, the qRT-PCR assay was conducted for ten selected uni-genes that included unknown genes from the uni-transcript (Table 12). RNA-Seq prediction reported uni-transcript sequence 1_ CL2766Contig1, 1_ CL1576Contig1, 1_ CL1679Contig1 and 1_ CL2484Contig1 as being downregulated while uni-transcript 1_ CL2708Contig1, 1_ CL41Contig6 and 1_ CL2624Contig1 were upregulated. As for two selected uni-genes with BLAST hits against homologous species other than *Gallus gallus*, 1_ CL1597Contig1 and 1_ CL2788Contig1 were selected as representatives. Expressions of these uni-genes from the RNA-Seq data were analysed by expressing the relative expression of each uni-gene into log_2_ fold-change. The log_2_ fold-change values were plotted into a standard curve. While performing the assay, two of the selected uni-genes; 1_CL2788Contig1 and 1_CL2766Contig1 were observed to not have significant amplification in the gradient PCR step, possibly due to the primers not being specific towards the selected sequences or absence of the sequences after extraction of the tissue. Hence, these were discarded from further analysed.

**Table 12:** List of uni-genes selected for validation using qRT-PCR. The respective differential expression and function; previously known or predicted through this study are listed accordingly to each uni-genes. *Up – Upregulated *Down – Downregulated

Uni-gene Name	DE	Function status	Function
1_CL1597Contig1	Up	Previously known	Interleukin 18 binding protein
1_CL2788Contig1	Up	Previously known	Extracellular matrix protein
1_CL2708Conti1	Down	Previously known	Cerebellar degenerative protein
1_CL41Contig6	Up	Previously unknown	Interleukin 18 binding protein-like or same pathway
1_CL2624Contig1	Up	Previously unknown	Interleukin 18 binding protein-like or same pathway
1_CL2766Contig1	Down	Previously unknown	Cell surface protein
1_CL1576Contig1	Down	Previously unknown	RNA binding protein Cerebellar degenerative protein e3 ubiquitin-protein ligase DNA replication licensing factor mcm7
1_CL1697Contig1	Down	Previously Unknown	Cell surface protein
1_CL2484Contig1	Down	Previously Unknown	Nicotinamide ribose kinase rbk2 Ubiquitin-conjugating enzyme e2c

The Cq values obtained from the qRT-PCR were calculated using the ∆∆Cq formula mentioned and the calculations presented in Table 13. As for the overall results, four uni-genes showed a positive %KD, indicating a downregulation profile; 1_CL1597Contig1 (82%), 1_CL2708Contig1(80%), 1_CL1576Contig1 (99%) and 1_CL2484Contig1 (99%). On the other hand, two uni-genes; 1_CL41Contig6 and 1_CL2624Contig1 had negative %KD values, which showed that these uni-genes were upregulated during the IBDV-infection in the host. Equally important, the FoxP3 and 1_CL1679Contig1 results displayed no amplification or expression during the infection state. The predicted downregulated uni-genes indeed were observed to be downregulated in vivo and similarly, the predicted upregulated in silico uni-genes were also seen to be upregulated. In sum, our RNA-Seq results were confirmed by the consistency between the qRT-PCR results and the RNA-Seq analyses.

**Table 13:** ∆∆Cq calculation performed on ten uni-gene sequences that were selected for validation. The six sequences were the unknown sequences (two upregulated and four downregulated), one was the FoxP3 gene and three sequences that had BLAST hits homologous to other species. Uni-gene 1 _CL2788Contig1 and 1_CL2766Contig1 were not reported as those uni-genes were discarded halfway through the qRT-PCR analysis. The average of GAPDH Cq is used to normalise the target gene expression *Exprs - Expression

Days P.I.	Cq of GAPDH (REF)	Uni-gene Name	Cq of Gene (TAR)	∆Cq	∆Cq Exps	Average ∆Cq Exps	∆Cq Exprs SD	∆∆Cq	∆∆Cq SD	KD%
Day 3	22.54	**1_CL1597Contig1**	31	8.04	0.003	0.002	0.001453	0.171016	0.090154	82%
	22.86		32.13	9.17	0.001					
	22.91	**1_CL2708Contig1**	30.03	7.07	0.007	0.006	0.001648	0.196888	0.051999	80%
	22.95		30.58	7.62	0.005					
	23.08	**FoxP3(1_CL2412Contig1**	**NO EXPRESSION**							
	23.40									
	23.11	**1_CL41Contig6**	31.40	8.44	0.002	1,973,293.77	2,790,659	7.586422	10.72882	**-VE VALUE**
	22.82		1.04	− 21.91	3,946,587.54					
	22.88	**1_CL2624Contig1**	37.25	14.29	0.00	1,493,899.42	2,112,693	9,999,717	14,141,736	**-VE VALUE**
	23.24		1.44	−21.51	2,987,798.85					
	23.29	**1_CL1576Contig**	37.23	14.27	0.00	0.000	8.55E-05	0.000291	0.000224	99%
	23.51		35.46	12.51	0.00					
	22.99	**1_CL1679Contig1**	**NO EXPRESSION**							
	22.84									
	23.12	**1_CL2484Contig1**	36.15	13.19	0.00	0.000	7.3E-05	0.005133	0.002365	99%
	22.44		35.17	12.21	0.00					
	22.56									
	22.64									
**AVERAGE (REF)**	22.95

**Table 14:** Oligonucleotides primer-probes designed for the ten uni-genes of interest amplification and validation. *fwd = forward strand, rev = reverse strand

Uni-genes	Primer Sequence	Melting Temperature (^**0**^c)
1_CL1597Contig1 (fwd)	GTG AAG TTG GTG CTC AGG TC	59
1_CL1597Contig1 (rev)	GCT CTA CTG GTT GGG AAA CG	
1_CL2788Contig1 (fwd)	TGA CGT TGT GCA CTT CTT GG	–
1_CL2788Contig1 (rev)	GTG AAG ACG AGG CAG CAC	
1_CL2708Contig1 (fwd)	CTT CTA GTT CGG TGT TGC GG	56.6
1_CL2708Contig1 (rev)	CGA GAG AAG GGC GCG ATG	
1_CL2412Contig1 (FoxP3) (fwd)	GGC AGA AAG CAC TT AGG TC	56.8
1_CL2412Contig1(FoxP3) (rev)	CAG CCG TAT GTT CGG GTA CT	
1_CL41Contig6 (fwd)	GGA GGA CAG TTG TAG GGA CA	55.7
1_CL41Contig6 (rev)	GTT GTC ACC CAC TGC GTG	
1_CL2624Contig1 (fwd)	TCC GCC GGC TTT AAT TCT TC	55.7
1_CL2624Contig1 (rev)	GCG GCG GGG AGA ATT AAT AA	
1_CL2766Contig1(fwd)	GCA CGT TCC CAT AGC TGT TG	–
1_CL2766Contig1(rev)	GAG GTG GAG CTG GA GTG AT	
1_CL1576Contig1(fwd)	GTG ATG GGT GTT GTG CTC AG	56.8
1_CL1576Contig1(rev)	AAG AAG AGA AA GAG GCC GT	
1_CL1679Contig1(fwd)	CCT GAG CCA TGA ATG ATA CGC	56.8
1_CL1679Contig1(rev)	GAC GAG GTGAAGGAG TCG AA	
1_CL2484Contig1(fwd)	AGG AGG AGG AGG AAG GCT AT	57.0
1_CL2484Contig1 (rev)	CCC TGT GAT GAG GTT TGC AG	

## Discussion

The constant occurrence of IBDV despite precautionary measures is one of the major concerns in the poultry industry. Some of the most virulent strains present in the world are present in Malaysia [26]. Vaccination has little effect on the progeny of immunised chicken flocks as studies have shown that there is no correlation between resistance or susceptibility of IBDV infection and the maternally-derived antibodies in chicks [3]. Also, the conventional vaccine commercially used for chicken immunisation against IBDV has been reported to lack in providing full protection while inducing new variant strains [8]. This is a major concern for the poultry industry worldwide as new strains may have more robust pathogenicity than the current strains. Hence, research studies regarding viral diagnosis, new vaccines and treatment methods to curb this disease are done globally and intensively. Thus, we performed de novo transcriptome assembly and compared the gene expressions of control and IBDV-infected chickens in six different inbred lines. In this study, we evaluated and discussed the functional significance of the observed variations in expressions of genes, whether up- or downregulated, following 3-days p.i. of IBDV. Additionally, we also studied the potential functional annotations of the unknown sequences, with the hope to meliorate the transcriptomics annotation of *Gallus gallus*. However, it is important to note that RNA expression does not directly correspond to the actual translated protein in the host. This study focused on the unmapped reads during the mapping of transcriptomes against the reference genome, as described in previous study of the group. The unmapped reads were assembled and clustered together, producing 10,828 uni-transcripts with low sequence redundancy. We explored the uni-transcript sequences produced from responses of six different inbred chicken lines towards IBDV infections to further decipher the transcriptomics activities and molecular changes during infection. The lines were used to perform the differential expression analysis of selected uni-genes. Currently, no previous documentation on the 12 up- and 18 downregulated uni-genes we studied had been done. However, it is equally important to note that a newly updated version of the chicken genome is available at Gallus_gallus-5.0 (GCA_000002315.5). Nevertheless, our findings showed that our de novo *Gallus gallus* dataset represented an important transcriptome resources for functional analysis and gene discoveries, as very few sequences had BLAST hits to the chicken genome.

Previously, resistant genes were predicted by analysing global transcriptomic differences in organs like the spleen and bursa, and between chicken lines that differed in susceptibility [44]. Genes involved in the extrinsic apoptosis pathway and the Toll-like receptor-signalling pathway, which played integral roles during the innate immune response were observed to be upregulated [44]. Genes involved in I interferon (IFN) response, pro-apoptotic cells, pro-inflammatory cytokines and chemokines, were also displayed to be upregulated in the bursal tissue, assumed to be due to the infected B-cells of the host [8]. In line with the known pathogenesis of IBD disease, S100A10 upregulation agreed with the notion of increasing cytokine and chemokines at the infected bursa tissue. Mechanistically, S100A10 would regulate the macrophage inflammatory immune responses [24]. Similarly, the upregulation of ccaat enhancer-binding beta protein (CEBPB) was reported to play an important role in cellular proliferation and transcription factor regulating immune and inflammatory responses gene expressions, especially in activated macrophages [21]. Meanwhile, Mucin-13 isoform XI (MUC13) was expected to be upregulated due to its function in cellular signalling at the haematopoietic transmembrane, enhancing cell-cell communications. Previously, MUC13 was reported to play a vital role in regulating inflammation, apoptosis and inhibiting infectious invasion [34]. Next, extracellular fatty acid-binding protein (EXFABP) upregulation was expected especially in chick embryo. It is annotated in the UniProt database to be highly important during young chickens’ development for innate immunity preparation and development [7]. Interestingly, IL-18 binding protein (IL18BP) annotated as a natural receptor antagonist of IL-18 which neutralised all IL-18 activities was also upregulated [10]. We assume an upregulation of IL18BP in IBDV-infected BF of chickens as a self-negative regulation mechanism to reduce inflammation inside the bursal tissue as targeting IL-18 with IL18BP is an achievable treatment for autoimmune disease in humans. However, IL18 presence will also further mediates the host’s innate immune response [9]., Nevertheless, the increase of Homeobox 1 expression in IBDV-infected BF remains elusive and yet to be understood in relation to viral infection.

It had been established based on other studies, specifically in the BF of chickens that genes involved in the B-cell receptor signalling and cell cycle pathways were dramatically downregulated following an IBDV-infection [16, 36, 38]. Notably, most biological responses observed in the chicken bursa tissues are widely accepted to be due to viral replication and cell damage and not due to antiviral responses [44, 45]. IBDV infection is widely known to suppress cellular proteins involved in ubiquitin-mediated protein degradation, energy metabolism, intermediate filaments, host translational apparatus and signal transduction [55]. Additionally, DNA replication licensing factors mcm7 (MCM7) and DNA-directed RNA Polymerase II subunit rpb1 (RPB1) downregulation were expected as both were reported to involved in DNA replication and cell differentiation activities [50]. Previously, RNA-binding protein 38 (RBM38) was reported to be involved in regulating the expressions of proteins of Parvovirus B19 vital to facilitate viral DNA replication [14]. This knowledge are worth mentioning as the vital reasons for the suppression of the mentioned proteins possibly due to the reducing number of dividing lymphocytes of B-cells or an activity in BF to suppress viral DNA replication. Other than the obvious suppression of expression-related activities, genes with cellular signalling-associated functions between cells for communication according to UniProt were also screened and annotated to reduce. B-cell receptor CD22 isoform XII (CD22) which aided in IgM- or CD4-binding, and the cell surface protein (CSP) was reported to be downregulated in all susceptible chicken lines in our study. Likewise, for Nicotinamide riboside kinase 2 (NMRK2), GMP reductase 1 (GMPR), Ubiquitin-conjugating enzyme e2c (UBE2C), RING-type E3 ubiquitin transferase uhrf1 (UHRF1), Aurora kinase b (AURKB) with annotated GO molecular function in ATP binding and metal ion binding categories, downregulation might be due to the suppression of cellular activities in infected tissues. Meanwhile, the sterile alpha motif-domain-containing protein 11 isoform XII (SAMD11) plays a vital role in the negative regulation of transcription via histone binding in IBDV-infected bursal tissues [19]. Suppression of SAMD11 expression further highlighted an increase of transcription regulation activities in infected BF. Lastly, the cerebellar degeneration-related protein 2 (CDR2) is known to have characteristics for aiding the viral proliferation downregulated profile exhibited by CDR2. This suggested that the chicken’s molecular response to control the viral infection was by inhibiting CRD2 expression during the IBDV-infected condition.

Briefly, both the upregulated and the downregulated uni-genes identified coincided with the pathophysiology of the disease. During an infection, genes involved in tissue development, necrosis and mortality have major contributions to disease pathogenesis. Among the upregulated genes, those involved in signalling from several cytokine receptors as well as in apoptosis, are differentially expressed. However, genes that are involved in endothelial cell development, proliferation and migration are downregulated [44]. The previously 12 upregulated unknown genes were also involved in cell signalling, cellular proliferation and differentiation while the 18 downregulated unknown uni-genes were seen to possess functions like cellular signalling, adhesion, and apoptosis. Remarkably, one protein, known to have functions for aiding viral proteins, the Cerebellar Degeneration-related Protein 2 (CDR2) was found in silico to be downregulated suggesting that this could be an immune response of the infected chicken against viral infection.

In our study, we have employed a transcriptomic approach to identify de novo genes from our unmapped reads. The RNA-Seq method was able to provide amazing and ground-breaking details about the transcriptional landscape. Although RNA-Seq technology is a highly efficient method to retrieve transcriptomic profiles in a short period of time, a small probability of false positive errors can occur. In order to increase the sensitivity of the data, our experimental design involved remapping of the unmapped reads to the de novo assembled and clustered uni-transcripts. Besides that, our study had also employed tools such as AUGUSTUS, MATCH and WGCNA R to perform integrated analyses of the de novo uni-genes. The applications of AUGUSTUS and MATCH analyses provided answers regarding the features of our novel uni-gene sequence structures and predict their completeness despite returning with negative matches and reports. Subsequently, although WGCNA output is not easy to be interpreted, we strongly believe that the application of it gives greater insights to the functional predictions of our data. Besides, it gives a critical advantage when relying on other common toolkits as the R package allow researchers to have control over the process of analysis, unlike other software. Hence, our experimental design produces a very valuable study on the comprehensive genomic or transcriptomic regulation mechanism of parasite infections on a host. This in silico method may provide the basis for in vivo or in vitro investigations, especially regarding the gene expression portfolio. Regardless of its benefits, limitation issues such as sequencing errors during cDNA synthesis, pre-processing stage, primers design errors and lack of the desired database may discourage researchers from pursuing this method. Nevertheless, the findings from our study are valuable assets in the quest to produce vaccines with high protection against IBDV, especially in understanding the molecular biological changes during infection.

## Conclusion

Apart from the attempts to control the disease through vaccines, understanding the chicken’s defence mechanism could aid in understanding the resistance or the susceptibility to the virus. To achieve this, the complete genome of the chicken needs to be studied. The *Gallus gallus* known annotated genome is stated as being complete. However, from our study, there are approximate ~ 10% of the genome yet to be discovered. The investigation of molecular changes in IBDV-infected *Gallus gallus* is one of the ongoing works in the field. In the present study, we comprehensively described the transcriptional responses of *Gallus gallus* Bursa of Fabricius following 3-days p.i. of IBDV. Overall, 10,828 uni-transcripts managed to be assembled from unmapped reads. A total of 618 non-redundant DE uni-genes was obtained using the RNA-Seq data, including 12 commonly upregulated DE uni-genes and 18 commonly downregulated DE uni-genes. Among the commonly DE uni-genes, three upregulated and five downregulated DE uni-genes did not have homologues in the NR (protein) or Swiss-Prot databases. Thus, we decided to utilise network analysis tools. Evidently, the ‘network analysis’ tools indeed helped in the prediction of the functions of the mentioned unknown uni-transcript sequences. The sequences were grouped into modules based on the correlation coefficients calculated based on a soft power score. The module results indicated that the sequences with no BLAST hits could either be part of the same pathway or a functional group, thus, aiding in predicting the function of the unknown sequences. The identified uni-genes in our studies were mostly classified as genes related to immune-related or cellular signalling activities. The model established by comparing the differences in gene expressions of IBDV-infected and control chickens could promote further studies for addressing the molecular mechanisms underlying the pathophysiology of the disease. The results of this study can be further analysed to look for viral particles in the RNA-Seq data and to generate any correlation between viral particles and genes from the chicken host, with the purpose of throwing more light on the pathogenicity of IBDV in chickens.

## Materials and methods

### vvIBDV infection in chickens

The inbred SPF chicken trial was carried out according to the guidance and regulations of the UK Home Office under the provisions of the project license no. 30/3196 issued by the Secretary of the State, Her Majesty’s Government of the United Kingdom [11]. Six flocks of unvaccinated White Leghorn inbred chicken lines; 15, 6, 7, N, O and P were supplied by the Institute of Animal Health, Compton. The percentage heterozygosity of the lines are 25.6% (N), 19.8% (O), 30.2% (P), 4.4% (6), 10.5% (7) and 13.3% (15). The inbred lines have been maintained by full-sibling mating through over 20 generations. The inbred lines with mixed genders were randomly assigned into two groups; infected or control experimental groups. Seven birds from lines N, 6 and 7, six birds from line P and five birds from lines O and 15, were infected via the intra- with vvIBDV strain UK661 of 105.4 EID50 via the intra-nasal route at age of 4–5 weeks. Age-matched and infected control birds were housed in a separate experimental animal room and provided with both vegetable-based feed ad libitum and water. Meanwhile, birds from the control groups (only three birds per line) were mock-infected with Phosphate-buffered saline (PBS). The validation of novel predicted genes in the control and IBDV infected samples of SPF chickens was carried out according to the Institutional Animal Care and Use Committee of the Faculty Veterinary Medicine (FVM), Universiti Putra Malaysia (UPM) (reference number: UPM/IACUC/AUP-R051/2014) [12].

### Tissue collection

Bursa of Fabricius (BF) tissues from the inbred chicken lines was harvested following the infection. The bursa tissues were collected from the control groups (one pooled sample per line) and the infected groups (two pooled samples per chicken line) tissue collection is at 3-days p.i. On the sampling day at 3-days p.i., the birds were euthanised by cervical dislocation and the gross changes of the bursal were collected and examined. Meanwhile, the remaining bursa tissues from each line were kept in RNAlater (Ambion, UK) and later, shipped to Laboratory of Vaccine and Immunotherapeutics, Institute of Bioscience, UPM. A total of 18 samples collected (three samples per chicken line) were sent to a sequencing facility to produce the transcriptomics raw reads via RNA-Seq.

### RNA isolation

The RNA preparation method was used by a previous group [11, 12]. Briefly. six bursal tissues samples from the control group and 12 tissues samples from the infected group were subjected to RNA-Seq. Notably, the two samples from the infected chicken group for all inbred lines were named infec1 and infec2. The number of transcriptomes decided was within the general consensus recommended for RNA-Seq analysis [41]. 20–30 mg of bursal tissues of each sample were homogenised using the Tissue Ruptor (QIAGEN, UK) in the presence of RLT lysis buffer (QIAGEN, USA). The total RNA was isolated using the RNeasy Plus Mini Kit (QIAGEN, UK) according to the protocol provided by the manufacturer. Using the Bio-Spectrophotometer (Eppendorf, USA) and Bioanalyzer 2100 (Agilent Technologies, USA), the RNA concentration and purity. Purified RNA samples at A260:A280 ratio of 1.8 with integrity number > 8.00 were used for the RNA sequencing.

### RNA-Seq for cDNA synthesis

**Fig. 10:**
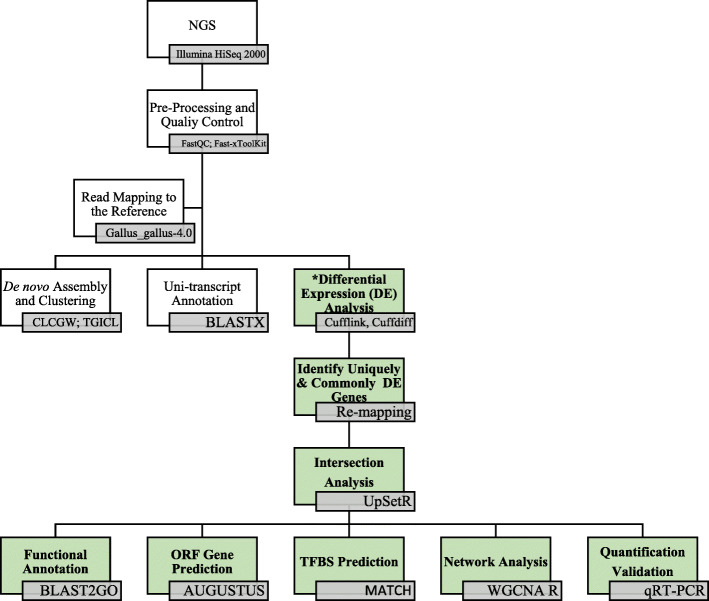
Overall workflow of the experimental setup and bioinformatic analysis performed in this study. Transcriptomic data obtained from NGS were subjected to statistical analysis and quality control prior to functional annotation. Then, the pre-processed transcriptomes were mapped to the reference genome. Unmapped reads against the reference genome were assembled and clustered to produce uni-transcripts data. The newly generated data; uni-transcript were subjected to differential expression analysis that included steps towards novel gene discovery via BLAST2GO enrichment analysis, prediction of ORF gene and TFBS, as well as gene network analysis. Lastly, the selected novel genes identified were determined to perform quantification validation

The isolated RNA from the collected BF tissues were selected accordingly to its parameter that could produce high quality reads before the cDNA synthesis process. The cDNA libraries of the 18 transcriptomes were generated using Illumina HiSeq 2000 paired-end type. Reaction mixture used for cDNA conversion using the SensiFAST cDNA conversion kit were followed accordingly to the manufacturer’s guideline. The output of the preparation resulted in a sequence of each of the samples that consists of more than 50 million raw reads of > 4.6 GB of sequencing data. These reads were further subjected to the typical downstream processes that include (i) pre-processing of sequencing reads, (ii) read mapping to reference genome, (iii) de novo assembly of unmapped reads and (iv) expression and differential expression analysis [27]. This study only focuses on the de novo assembly and the uni-transcripts generated through this assembly. The overall workflow is represented in Fig. 10.

**Table 15:** The Knockdown Percentage (KD%) calculation for the RNA-Seq validation method

**Step 1**	
Normalize to (REF): ∆Cq = Cq (TAR) – Cq (REF)	
**Step 2**	
Exponential expression transform: ∆Cq Expression = 2–∆Cq	
**Step 3**	
Averages replicates and calculate standard deviation	
**Step 4**	
Normalize to treatment control	
Step 5. % KD = (1 – ∆∆Cq) × 100	

### Read processing

RNA-Seq raw reads were subjected to quality assessment and pre-processing to check the read quality, using FastQC v0.10.0 [1] and FASTX-Toolkit v0.0.13 [15]. To ensure that the FASTQ reads were of good quality, the first 13 bases from the 5′ end were removed due to ambiguous GC content. Low quality bases (Q < 20) were also trimmed off. Finally, reads that were shorter than 30 bp or those that contained unknown bases (N) were discarded. The total number of reads from each sample before and after pre-processing was as in our transcriptome profiling paper (REFERENCE). As most of the reads were of good quality, more than 99.9% of the reads passed the quality threshold and were used for further analyses.

### Sequence read mapping to reference genome and external RNA controls consortium (ERCC) library

Pre-processed reads obtained from each sample were previously mapped to the reference genome Gallus_gallus-4.0 from NCBI (GCA_000002315.2) and the sequences of ERCC spike-in (Ambion ERCC ExFold RNA Spike-In Control Mixes [Cat. No. 4456739]). The ERCC spike-in value was log_2_-transformed and plotted as a dose-response curve and R^2^ was determined from known ERCC transcript number in relation to the read density output (fragments per kilobase of transcript per million mapped reads, FPKM). It served as an assessment for the RNA-Seq platform performances as described in the manufacturer’s guidelines. Mapped read is the term used for reads with local alignment with the reference sequence, while uniquely mapped reads are reads that mapped to only one region of the reference sequence. The statistical report of the read mappings was observed for further decision of the next process before the next phase.

### Sequences read mapping to Uni-transcripts

Due to the presence of unmapped reads from the previous study, these unmapped reads were later mapped to the uni-transcripts that were built from the de novo transcript assembly process (see the De novo transcript assembly method below). The read mapping of unmapped reads onto uni-transcripts were carried out using TopHat v2.0.6 [47], which used Bowtie v2.0.0-beta7 [23] as its algorithmic core, by allowing two reads mismatches and strand-specific processing of reads. The Bowtie software served as a statistical quality assessment of the assembled uni-transcripts to avoid overestimation of transcriptomics quality during the read mapping process. Both sequences mapping methods produced quantitative statistical evidence on the accuracy and reliability of our reads in this study.

### De novo assembly and transcripts clustering

The 2900–4300 transcripts unmapped reads that did not map to the reference genome from the previous finding were de novo assembled for each sample using single-threaded Velvet v.1.2.08 [53] followed by Oases v.0.1.22 [40]. The assembly was also performed by CLC Genomics Workbench by QIAGEN Bioinformatics v6 [35] and the results were comparable. A range of k-mer size (45–71) was tested for each sample and the best k-mer size to achieve the highest N50 value (k-mer size of 57 ~ 63) was applied for the samples. The strands-specific parameter was switched off before processing, to increase the coverage of the assembly by using as many reads a possible to build the assembly. After the assembly, transcripts with less than 100 bases were discarded.

Then, assembled transcripts from all the samples were iteratively clustered according to their similarity and pooled together using TIGR Gene Indices Clustering Tools (TGICL) [32] producing a non-redundant set of uni-transcripts with the fewest sequence redundancy. The transcript clustering parameters were set as 96% minimum identity for overlaps, 30 bp minimum overlap length and 30 bp maximum length of unmatched overhangs. The output assembly from the mentioned clustering tool was named as ‘uni-transcripts’ for this study. The statistical details report such as the total number of uni-transcripts, the total size of uni-transcripts (bp), N50 stats (bp) and GC% were checked before further used for the unmapped reads mapping process (mentioned above). The read mapping results were also used to decide the sequence direction of each uni-transcript (strand with higher expression level was assigned as sense strand).

### Uni-transcripts annotation

The uni-transcripts generated were searched against NCBI non-redundant (NR) and Swiss-Prot database by using BLASTX with expect value E-value ≤1.0e-5. The top 20 non-redundant (nr) results and the top 20 Swiss-Prot results, which had the lowest E-value and the highest coverage were analysed, using BLAST2GO v2.6.3 [5] for Gene Ontology (GO) annotations. The results reported relevant information such as the number of uni-transcripts with hits or with no hits, top-hit species distribution and GO categories distribution.

Then, BLAST2GO was again used, but inserted with a selected set of uni-genes sequences that were reported to be commonly up- or downregulated in all six inbred lines. The two sets of data were analysed separately using hits information generated by the databases. Information from BLAST2GO such as Top-hit species distribution and molecular functions were used to further analysed our de novo findings.

### Uni-transcripts expression and differential expression analysis

The analyses of uni-transcripts expression and differential expression was done using Cufflink v2.0.2 and Cuffdiff v2.0.2 [48, 49]. This analysis was carried out by keeping the strand-specific parameters turned on. The expression levels of each gene or uni-transcript were expressed as FPKM with a cut-off FPKM> 1.0e-5 (determined from the ERCC dose-response analysis). In this study, differentially expressed uni-transcripts are defined as uni-genes (previously known as uni-transcript sequences, henceforth called as uni-genes for brevity), with log_2_ fold-change<− 2 or log_2_ fold-change> 2 between the infected and mock-infected samples with a false discovery rate of q-value < 0.05. Meanwhile, differentially expressed uni-transcripts which showed expression only in one condition; either mock-infected, infected or control, with the q-value < 0.05 were considered uniquely differentially expressed uni-genes.

### Uni-genes intersection analysis

UpSetR [6] is a package that allows visualisation of intersections and their size, suitable for data with many groups. The differentially expressed sequence IDs from all six lines were taken as inputs and used to generate two differential expressions uni-genes analysis, each representing the upregulated and downregulated sequences, respectively. Firstly, the most significant uniquely mapped sequences against uni-transcripts were short-listed into a table, by listing out uni-transcripts with the least redundancy and statistically significance with *p* < 0.05. Using UpSetR [6] package, the commonly upregulated and downregulated sequences seen in all six inbred lines were emphasised with red (for upregulated) and blue (for downregulated). The visualised UpSet plots will be used to understand the gene expression interactions between control and IBDV-infected of Bursa of Fabricius.

### Gene prediction on commonly differentially expressed genes with no BLAST hits analysis

Uni-genes sequences with no BLAST hits from the BLAST2GO step were then subjected to AUGUSTUS (gene prediction software) [46]. This tool is used to predict potential open reading frames (ORFs) based on the Hidden Markov Model. Three sequences from the upregulated list and four sequences in the downregulated list without BLAST hits were subjected to the gene prediction analysis.

Next, the uni-genes sequences with no BLAST hits were further employed onto geneXplain’s MATCH programs [20]. MATCH is a tool programmed to predict transcription factor binding sites in DNA sequences via weight matrix-based calculation and used the library from TRANSFAC® Public 6.0 [52]. Only high quality matrices with the most minimal false positive matches were cut-off as the output.

### Gene function prediction

#### Weighted gene correlation network analysis (WGCNA)

Co-expression analysis was performed to identify modules of highly correlated genes [54]. WGCNA is a concept of converting co-expression measures into correlation weights and nodes which will create genes co-expression networks to understand the interactions between genes. The gene expression FPKM values of the uni-genes from Differential Expression Genes analysis were log_2_-transformed before being processed through the WGCNA R tool package [22]. Different in-built functions had been used to select the best parameters (soft power = 14) and the threshold of the co-expression module was set to *p*-value < 0.05. All other parameters were set at default values.

### Validation of novel predicted genes

#### RNA extraction, cDNA synthesis and real-time quantitative PCR assay (qRT-PCR)

Using the bursal tissue collected, the total RNA from the control and infected samples was isolated using a RNeasy Plus Mini Kit (QIAGEN, UK) according to the manufacturer’s guidelines. The RNA concentration and purity were measured using a Bio-Spectrophotometer (Eppendorf, USA). One μg of RNA sample was reverse transcribed into cDNA using a SensiFAST cDNA Synthesis Kit (Bioline, USA) in a 20 μL reaction mixture. The cDNA synthesis reaction was performed in a thermal cycler (Bio-Rad, USA) with the following cycle profile: primer annealing at 25 °C for 10 min, reverse transcription at 42 °C for 15 min, inactivation at 85 °C for 5 min, and finally, hold or chill at 4°c. Gene expression in the bursal tissue was analysed using Custom TaqMan Gene Expression Assays (Applied Biosystems, USA) with specific primers and probes targeting the ten selected uni-genes (Table 13); three commonly upregulated and four commonly downregulated unknown genes, FoxP3 gene, and two genes that had BLAST hits against homologs of species other than *Gallus gallus.* Prior to quantification, total RNA extracted from the vvIBDV-infected bursal tissues was used as a positive control to generate a standard curve for each gene in a tenfold dilution series ranging from 1000 ng/reaction to 0.1 ng/reaction. Each gene was assayed in triplicate using a CFX96 real-time system (Bio-Rad, USA), with the following cycle profile: one cycle at 50 °C for 2 min and one cycle of 95 °C for 10 min, followed by 40 cycles at 95 °C for 15 s and 60 °C for 1 min. For the quantification of gene expression, each cDNA sample was assayed in triplicate and the expression value was normalized using a reference gene: Glyceraldehyde 3 Phosphate Dehydrogenase (GAPDH). Differential gene expression was expressed as the log_2_ fold change relative to the uninfected control using the -∆∆CT method (Table 2).

## Data Availability

The datasets of sequences used in supporting this article this study are available in the European Nucleotide Archive (ENA) with the study no. PRJEB9318, [http://www.ncbi.nlm.nih.gov/bioproject/?term=PRJEB9318].

## Abbreviations

IBD: Infectious Bursal Disease
IBDV: Infectious Bursal Disease Virus
vv: very virulent
BF: Bursal of Fabricius
RNA-Seq: RNA-Sequencing
DE: Differentially expressed
GO: Gene Ontology
qRT-PCR: Quantification Real-Time Polymerase Chain Reaction
